# Factors influencing long-term medication non-adherence among diabetes and hypertensive patients in Ghana: A qualitative investigation

**DOI:** 10.1371/journal.pone.0193995

**Published:** 2018-03-28

**Authors:** Roger A. Atinga, Lily Yarney, Narissa Minta Gavu

**Affiliations:** 1 Department of Public Administration and Health Services Management, University of Ghana Business School, Legon, Accra, Ghana; 2 Medical Department, Korle-Bu Teaching Hospital, Accra, Ghana; Florida International University Herbert Wertheim College of Medicine, UNITED STATES

## Abstract

**Background:**

Evidence remains limited on why diabetes and hypertensive patients admitted to long-term drug therapy miss doses or discontinue medication taking. We examined this phenomenon from the perspective of diabetes and hypertension patients at a Ghanaian teaching hospital.

**Methods:**

Between July and December 2015, we conducted a qualitative study targeting caregivers and their patients with chronic diabetes and hypertensive on re-admission at the Korle Bu Teaching Hospital due to non-adherence to prescribed medication. Participants were sampled purposefully and taking through in-depth interviews using an interview guide. Notes and audio recordings of interviews were transcribed, managed and coded for themes guided by the thematic network analysis recommended by Attride-Stirling.

**Results:**

Non-adherence was the result of perceptions that the medications are not effective for managing the conditions. Patients with these perceptions rejected the medications and turned to herbal medicines and spiritual healing as therapeutic alternatives, because of their easy accessibility, perceived efficacy and affordability. Other factors identified to influence non-adherence included polypharmacy practice; tight work schedules; social norms; poor prescription instruction by health providers; and knowledge and experience of medication.

**Conclusion:**

Findings suggests the need for health providers to adopt therapeutic approaches that take into account patients’ beliefs, values and norms in administering medications. Sensitisation of patients and caregivers during admission on the implication of non-adherence, as well as interventions that monitor and provide feedback mechanisms on patients’ medication taking behaviour holds promise for maximising diabetes and hypertensive medication adherence.

## Introduction

Diabetes and hypertension are the highest burden of non-communicable diseases to populations worldwide [1, 2]. An estimated 366 million and 1 billion people globally are living with diabetes and hypertension respectively [3, 4]. By 2020, prevalence of these diseases is expected to increase between 13% and 30% [1, 2], without more innovative preventive interventions. Diabetes and hypertension prevalence are increasingly high in Low-and Middle- Income-Countries (LMICs) that are experiencing rapid transition in health and diseases patterns, and their interaction with the social determinants of health [5]. Governments in LMICs countries have over the years increased investment on interventions to improve health systems responsiveness as a vehicle to control and reduce the health, social and economic burden of these non-communicable diseases [6, 7]. Such investments, however, are undermined by persistent unhealthy lifestyle choices including tobacco use, poor diet and physical inactivity [5, 8].

Awareness of risk factors and preventive measures for diabetes and hypertension is low in LMICs compared to high income regions [8, 9]. In some countries, physical and financial access to appropriate diagnostics for high risk populations is a challenge as is treatment medication for affected individuals [10, 11]. Even if individuals are diagnosed and admitted to long-term drug therapy essential for managing diabetes and hypertension, poor adherence to prescription often pose as a barrier to realising effective therapeutic outcomes [12].

Adherence to drug therapy describe the extent of a person’s behaviour in terms of taking drugs or executing life style changes in conformity with health provider recommendations [13]. Non-adherence arises when a patient irregularly follow prescription or discontinue use [14], for reasons either intended or unintended [15]. Non-adherence often increases the risk of relapse, poor therapeutic outcomes and needless mortalities [14]. Hospital admissions and readmissions, emergency outpatient visits and mortalities increases when patients poorly adhere to medication as directed [16, 17]. Increase hospital visits due to non-adherence in turn contributes to depletion of available scarce resources and supplies in health facilities [18].

Prevalence of medication non-adherence is on the ascendancy especially in LMICs where adherence averages less than 50% [13]. In Ghana as with many other LMICs, national level data on medication adherence particularly among diabetes and hypertension patients is sparse. Studies in two different settings, however, found 38.5% adherence rate among diabetes patients [19] and 47.7% for hypertensive patients [20]. These statistics are unacceptably lower than the recommended 90% threshold [21]. This makes it urgent and compelling to uncover the underlying factors influencing poor medication taking behaviour among these patient cohorts. Yet, studies drawing policy makers and practitioners’ attention to the wide ranging factors influencing non-adherence remains limited in Ghana.

A handful of quantitative studies have identified age, income, education and gender as demographic factors influencing non-adherence to malaria and diabetic drug treatment [19]. Such demographic analysis are crucial, but they tend to limit understanding of the complex set of factors that on their own or in interaction with others influence non-adherence in terms of skipping doses or discontinuation of meditation taking [22]. Also missing in existing studies is why non-adherence occur among hypertensive patients. To fill this research gap, this study qualitatively explored how and why chronic diabetic and hypertensive patients admitted to long-term drug therapy poorly adhered to prescribed medication taking.

## Methods

The study was conducted in the Korle-Bu Teaching Hospital located in Ghana’s capital, Accra, in the Greater Accra region. Starting from less than 200 beds and 200 outpatient visits at its inception in 1923 [23], the hospital has seen tremendous expansion over the years with about 2000 beds, 21 clinical and diagnostic departments and 3 Centres of Excellence. Average daily outpatient visits and inpatient admissions have increased to about 1500 and 250 respectively [24]. Korle Bu is the top referral point for patients within and across Ghana, especially West African Countries [25]. Chronic communicable and non-communicable diseases are often among the highest reported cases and associated mortalities in the hospital [26].

We targeted patients admitted to long-term diabetes and hypertension drug therapy in the hospital, but were subsequently non-adherent, either by skipping doses or discontinued with the medication taking [12], leading to relapse and readmission. In order to cross validate patients’ perspectives and also account for their limitations in providing narratives in more detail, we included their caregivers at the time of admission in the hospital. The study was qualitative, employing in-depth interviews using a discussion guide to explore factors that pertained but not limited to personal, family or community and health provider factors that may have influenced non-adherence. These factors were derived from a cursory review of existing literature on medication non-adherence [27, 28].

Interviews were held separately for patients and their caregivers during admission and/or the day of discharge depending on their request. Interviews took the form of discussions, using series of inductive probes to widen the narratives as much as possible. This process enabled the gathering of additional insightful factors not covered in the discussion guide. Notes were taken during interviews and if participants agreed they were audiotaped. Interviews were conducted in either English or Twi (a local language predominantly spoken in every part of Ghana) depending on participants choosing and lasted for an average of about 1 hour.

The team of data collectors comprised two of the authors (RAA and NMG) and one research assistant who was given a day’s training on qualitative methods. The team visited the hospital’s medical ward biweekly, each time being assisted by the ward nurses to purposively recruit participants into the study. In the first month of data collection, 4 patients and 2 caregivers were recruited. More or equal number of participants were subsequently recruited till thematic variations were observed [29]. At the end of the study period–July to December 2015, participants totalled 49; comprising 32 patients and 17 caregivers (Table 1).

**Table 1:** Numbers of participants recruited each month, July and December 2015.

	July	August	September	October	November	December	Total
Number of patients recruited	4	6	5	7	6	4	32
Number withdrawing	0	1	0	2	1	0	4
Number of care givers recruited	2	4	2	3	3	3	17

The study received approval by the Ghana Health Service. Verbal consent was sought from each participant. Prior to each interview, the study’s purpose and its potential contribution to improving medication adherence were explained. Next, patients and their caregivers were made aware that participation in the study was voluntary, with a further assurance of their right to withdraw from the interview at any time.

Audio recordings in the local language (Twi) were translated into English and crossed verified by the last author (NMG) who simultaneously compared the audios and transcripts. The transcripts together with the field notes were read thoroughly, refined and then exported into Nvivo version 11 for analysis by RAA (the first Author). To ensure rigour and accuracy of the software coding process, the transcripts were doubled coded by RAA and an independent person guided by Attride-Stirling [30] stages of thematic network analysis. In the first stage, we set out to breakdown the text by assigning codes to key words or phrases reflecting participants’ viewpoints on non-adherence–discontinuation of medication-taking, missing of doses or both. About 102 codes were derived at this stage based on their frequency of occurrence, but only 35 are reported. The second stage extracted basic themes briefly describing the codes generated from stage one. Basic themes were then represented by organising themes in the third stage. Finally, global themes were generated and agreed upon to give an overall meaning to the lower-order themes abstracted [30]. Table 2 illustrates the codes and themes building process. Results are presented based on the broad themes and verbatim quotes referenced as either hypertensive or diabetic patient, or comorbidity patient (those with both conditions).

**Table 2:** Codes and themes building process.

Codes	Basic themes	Organising themes	Global themes
➢ Efficacy	No sign of relief during medication-taking	Negative perception of medication	Perception of medication efficacy
➢ Trust	Medication not effective		
➢ Ineffective			
➢ Relief			
➢ Herbal medicine	Trust in herbal medicine effectiveness	Herbal medicine work better	Recourse to herbal medicine
➢ Available	Native doctors cures proximal causes of conditions		
➢ Affordable			
➢ Native doctors			
➢ Curses and witchcraft			
➢ Prayers	Recourse to healing through prayers and fasting	Spiritual healers offer effective therapy	Recourse to spiritual or divine healing
➢ Fasting and prayers	Spiritual healers have power to heal		
➢ God can cure			
➢ Spiritual healer			
➢ Spiritual healing			
➢ Experienced disorders	Purchase of over-the-counter drugs	Negative effect of regime combination	Interaction effect of polypharmacy practice
➢ Purchased drugs	Regime combination		
➢ Offered drugs from relatives			
➢ Additional drugs			
➢ Work	Routine work schedules	Heavy work demands	Work and routine busy schedules
➢ Poor memory	Frequent and overstayed travels		
➢ Forgetfulness			
➢ Overstayed travels			
➢ Work related travels			
➢ Not a serious condition	Social pressure to stop medication-taking	Family and community norms discouraging medication taking	Societal norms
➢ Derisive comments	Norms used to discourage medication taking		
➢ Medication not the solution			
➢ Understanding	Poor explanation of dosage	Self-dosing schedule due to poor instructions and explanation	Poor understanding of prescriber instruction
➢ Poor explanation ➢ Handwriting ➢ Instruction	Unclear instruction of medication taking		
➢ Side effects	Previous experience of medication	Fair idea of medication	Knowledge and experience of medication
➢ Unpleasant	Knowledge of medication side effect		
➢ knowledge			
➢ Experience			

## Results

### Participants’ characteristics

Table 3 shows that the majority of the participants were males (57.1%), aged 40 years and above (60.7%), employed (39.3%), Christians (53.6%) and currently married (64.3%). There was slightly more diabetes (39.3%) than hypertension (32.1%) patients, while 28.6% were suffering from both conditions.

**Table 3:** Characteristics of study participants.

**Gender**			
Male	Female		
28 (57.1%)	21 (42.9%)		
**Age**			
Below 39 years	40+ years	Mean (Std. dev.)	Range
19 (38.8%)	30 (61.2%)	42 (37.5)	31–57
**Employment**			
None	Employed	Retired	
16 (32.6%)	19 (38.8%)	14 (28.6%)	
**Religion**			
Christian	Muslim	Traditional African	
26 (53.1%)	14 (28.6%)	9 (18.4)	
**Marital status**			
Currently married	Currently single		
31 (63.3%)	18 (36.7%)		
**Condition**			
Hypertension	Diabetes	Both	
17 (34.7%)	19 (38.8%)	13 (26.6%)	

### Factors influencing diabetes and hypertension medication non-adherence

This section presents findings of the study on non-adherence to hypertension and diabetes medication taking. Findings are presented in line with the eight broad themes generated (Table 2) which include: perception of medication efficacy; recourse to herbal medicine; recourse to spiritual or divine healing; polypharmacy practice; tight work schedules; social norms; poor prescription instruction by health providers; and Knowledge and experience of medication.

### Perception of medication efficacy

Non-adherence stemmed from patients’ low trust in the medications efficacy. Patients were admitted to drugs such as Nifedipine, Methyldopa, Losartan and Lisinopril for hypertension; and Metformin and Insulin for diabetes. However, it was found some patients had poor perception of the medications’ efficacy in alleviating their conditions. The perception of not feeling much relief during the course of taking the medications further influenced decisions to discontinue:

“I remember I was given drugs like Methyldopa and some others but in the course of taking them I realised they were not helpful. I did not feel like having a relief, so I stopped.” (Female, hypertensive patient).

“I took the drugs for a while and stopped because they are not effective at all. I did not feel being relieved of the condition, so I thought it was better for me to stop.” (Female, comorbidity patient).

### Recourse to herbal medicine

Non-adherence was linked to recourse to traditional or herbal medicine. Some participants said they were under pressure from peers, family and relatives to take herbal medicine, because it is more secure, natural and efficacious.

“He used to take the drug regularly and I commended him for that. But after some time, he told me that his friend introduced him to natural mixtures which are effective than the prescribed drugs. Since then he stopped taking the medications.” (Female, Caregiver).

Some patients who resorted to herbal medicine complained about high cost of the prescribed medications. While some of them lamented that they economically weak to continue buying the drugs, others simply did not want to get used to the medications which they cannot afford. For them, herbal medicines are cheaper, affordable and available, hence the earlier they started with them the better.

“…since we cannot afford to buy the drugs always, I advised her to stop the drugs so that she does not get used to them. She agreed and we started with herbal medicine, but the sickness became worse and we rushed her here.” (Male, Caregiver).

Some patients felt their illness was not borne out of natural causes. They claimed curses, witchcraft and spells from family members were proximal causes. They believed herbal mixtures provided by native doctors were appropriate, because they have the power to cure these unnatural causes.

“He abandoned the drugs, because according to him, his sickness is associated with a curse by the grandfather. He said he preferred herbal medicine prepared by native doctors because such concoctions have the power to prevent everything causing the sickness.” (Female, Caregiver).

### Recourse to spiritual or divine healing

Patients discontinued with the medication-taking and resorted to spiritual or divine healing in the form of prayers, fasting and paranormal approaches. The belief was that prayers work better than medication because ill health is an act of God.

“You can’t take away the healing powers of prayers! Yes, I abandoned the drugs. God knows my problem, I will continue to pray until this sickness is gone.” (Female, diabetic patient).

There were others discontinued and held onto fasting and prayers; noting fasting heals because it deprives the body of proximal causes of the condition.

“Actually I was not taking the medicine because, I was fasting and praying for divine intervention. I was also fasting and praying to deprive myself of life style choices that causes the condition.” (Female, diabetic patient).

The belief that spiritual healers have the power to heal disease conditions influenced discontinuation of the medication-taking. Patients said they consulted spiritual healers believed to have successfully healed similar conditions and hoped to get well by their healing powers without the prescribed medications. Spiritual healers were also perceived as having the power to foresee and expel evil forces behind illness.

“I realised that there is nothing these spiritual healers cannot heal. I heard the miracles and healing powers they possessed and said to myself, if they can do all that then I will consult them for my problem. So I abandoned the drugs and went to them.” (Male, comorbidity patient).

Spiritual Healers way of healing including through ‘laying on of hands’ or distant healing through sacrifices and intercessory prayers influenced non-adherence as they waded in and discouraged patients from taking the medications.

“…The man of God told him that he was not getting well because he (man of God) was praying and making sacrifices to God on his behalf. So he should put his trust in him and do away with the medications, for God heals those who have faith in Him. Because of that he abandoned the drugs.” (Female, Caregiver).

### Interaction effect of polypharmacy practice

Patients mentioned that during the course of medication, they experienced disorders and mild severity of the illness. This led them to purchase a variety of over-the-counter drugs for relief. But as they experienced adverse reactions from the multiple regime combinations, they decided to discontinue with the prescribed medication. As stated by a caregiver:

“We thought that we were doing him a favour by buying him other drugs only to realise it was not worth it. As he started taking all the drugs made available to him, the condition was worsening. So we advised him to stop taking the prescribed medicine. Since then the conditions became more severe and that is why we came here.” (Female, Caregiver).

Patients also missed doses as a result of regime combinations that also include herbal medicines.

“He was not taking the medications regularly. He will take the medicine today, switch to other drugs or herbal mixtures and come back to it another time.” (Female, Caregiver).

### Routine work and related busy schedules

Statements by most of the participants suggested forgetfulness caused by routine schedules compromised medication schedules. Patients who had started taking the medications as directed later missed doses because of preoccupation with routine work, daily activities and other engagements.

“I normally wake up around 3.30am and by 4.00am, I leave the house for work. Sometimes you get to the worksite and realise that you don’t have the drugs with you. Because of that I missed most doses and that is why the sickness started again.” (Male, hypertension patient).

Patients who travelled frequently as required or mandated by their jobs were more exposed to forgetfulness, and therefore non-adherence. Such patients said they often forgot to carry the drugs in the baggage with them during pre-travel arrangements. And as they overstayed the travel, they discontinued taking the medication or missed doses.

“I am a driver and I travel with my boss frequently. Before my admission here, I forgot to carry the drugs with me during our trip and we stayed for more than three weeks. I eventually forgot that I was on medications till the sickness started again.” (Male, diabetic patient).

### Societal norms

Prevailing norms of the cultural and social environment were shown to influence patients’ discontinuation of the medication. Some participants stated that they were discouraged by relatives and family members who insisted it is against societal norm to resort to modern medicine to treat illness.

“Where I come from, people don’t believe in scientific medications at all. So when I returned from the hospital with the drugs, my relatives and family members encouraged me to abandon them because for how long will a young man like me continue to take medications.” (Male, hypertensive patient).

“As I started taking the drugs, my husband was not happy with that. He said, it is against the norm to take drugs simply because of hypertension. I initially took it for a joke but as he persisted I had to stop.” (Female, hypertension patient).

Few of the participants indicated that in their communities, beliefs and norms are used to trivialise certain morbidities including hypertension. They are not considered serious conditions that require modern medication to be healthy.

“…People were saying that hypertension is normal in life. It is not a serious condition and that everyone has it. So when she started taking the drugs, they said she is wasting her time and money. Because of that she stopped the drugs after taking them for some time.” (Female, Caregiver).

### Poor understanding of prescriber instructions

Concerns were raised about ambiguous and confusing explanations offered by health providers in regard to how the medications should be taken. Some patients misunderstood the explanations offered to mean that they can cease taking the medication once they felt better.

“…He talked for a long time and when I complained that the drugs are many, he told me that if that is the case I may stop once I am ok. So immediately I got better I stopped with the understanding that the illness is cured.” (Male, hypertension patient).

A few of the participants said health providers’ handwriting of the dosage was not decipherable enough. Therefore, they devised their own dosage regime above the recommended dosage, leading to complications. To avoid further worsening of the condition, they had to discontinue with the medication.

“I could not read the handwriting as to how the medication should be taken. When I asked others they gave me suggestions and I followed, but little did I know that it was over-dose. As I began to feel complications, I stopped taking the drugs.” (Female, hypertension patient).

### Knowledge and experience of medication

Knowledge of medications, especially among high literate patients greatly influenced non-adherence. Such patients were of the view that before taking the medication, they researched online and read the drug information leaflet to understand what the prescribed medication entail. They revealed that they got to know about side effects of the medications and that scared them.

I decided to read about the side effects before taking the drugs and what I read scarred me. I did not make any attempt to the take medicines because I didn’t want to go through the side effects.” (Male, hypertensive patient).

Some patients claimed that their research informed awareness that taking the medications could trigger other conditions; hence they discontinued in order to avoid such episodes.

“I read the instructions very well and realised that although the drugs are good, they will cause other conditions which are unpleasant. That is why I stopped.” (Male, diabetic patient).

Some patients cited unpleasant experiences such as side effects of the medicines as a reason for discontinuation.

“What I was going through while taking the medication was unpleasant. I was feeling uncomfortable and not as normal as I used to be. Because of that I advised myself and stopped taking the medication.” (Male, hypertensive patient).

## Discussion

We found non-adherence was the result of patients ill perception of medications efficacy, a finding that parallels others [28, 31], but contrasts with another involving patients from, and across Ghana, that showed perceived medication efficacy influenced non-adherence [32]. Patients also doubted efficacy of the medications and therefore did not have patience to follow the dosage till completion. We attribute this to common medication-taking behaviour in Ghana. A related study by Dodor and Afenyadub [33], showed that treatment default among TB patients had to with ill-feeling about drugs and the impatience to routinely take prescribed medication till completion. Such behaviours are usually the cause of poor outcomes leading to patients engaging in ‘healer shopping’ [34]. This finding suggest the need for patients to be properly counselled during dispensing to enable them appreciate the proven scientific efficacy of medications in the management of diabetes and hypertension. Health providers must stress that the benefits of medications are realised when patients carefully and regularly follow dosage regime.

The behaviour of patients refusing medication and turning to traditional herbal medicine is a growing phenomenon impeding orthodox care outcomes in Ghana [35]. Limited trust in, and perceived inefficacy of modern care usually account for such behaviours. A study in rural and urban Ghana, for example, showed that acute and chronic patients first sought modern care, but turned to traditional herbal medicine because of perception that modern medication is not effective [35]. The danger about herbal medicine is that their efficacy and toxicity has not been tested scientifically. Thus, users of traditional herbal medications are prone to developing viral resistance to medications and experiencing complications [36], as manifested in the results. The finding about patients resorting to traditional healers to revoke unnatural causes relating to sorcery and spells complements other studies in Ghana [37, 38], and suggests the need for interventions to pursue patients to rely solely on biomedical approaches for managing conditions. One such intervention is the suggestion by Aikins [34] that illnesses caused by belief systems should be treated with ethnomedical drugs scientifically approved to be nontoxigenic. Medical and ethnomedical practitioners can then collaborate to treat symptoms when necessary.

Non-adherence was associated with spiritual or divine healing through prayers, fasting and paranormal therapies. This findings reflects in the growing demand for spiritual healers services in treating conditions among segments of populations in Ghana [39, 40]. Patients usually prefer spiritual or divine healing because of its affordability and accessibility [41] as well as the belief that certain illnesses have spiritual causations beyond medical treatment [38]. The problem about spiritual or divine healing is the conflict with modern care [42]. This was brought to light as spiritual healers discouraged patients from taking the medications because it was incompatible with divine healing ways [41]. The belief that medication is unnecessary because ill-health is an act of God and uncontrollable except divine intervention supports Tabi *et al*. [38], whose study revealed individuals preferred healing by God to be the most safest.

The practice of polypharmacy leading to complications is a disturbing finding. In one breath, it reflects in irrational consumption of medications as a quick fix, when in fact the prescribed medications sufficed. On the other hand, it reflects in poor education given by providers on the consequences of consuming non-prescribed drugs while taking the medication. Whatever the problem, the fact remains that polypharmacy practice caused hazardous drug-drug and drug-disease interactions [43], resulting in patients discontinuing the medication-taking. This is in keeping with the finding that non-adherence increases with increasing consumption of more non-prescribed medications [44]. To avoid the risk of polypharmacy practice, patients and their caregivers should be educated on drug safety, and the importance of sticking to prescribed drug regime. Adverse drug effect due to polypharmacy should be explained and caution given to patients to report symptoms, rather than resort to self-medication with over-the-counter-drugs.

Cognitive problems relating to forgetfulness emerged as a barrier of adherence. Forgetfulness had underlying causes: routine work schedules and frequent travels, both of which disrupted daily medication schedule [45]. Among the factors that accounted for non-adherence, forgetfulness appears to be the most difficult to control. Because, its occurrence is involuntary. Despite this, we propose that patients exhibiting forgetfulness be given cognitive support. This can take the form of family members using cues to remind patients of daily medication schedules. Patients can also benefit from adherence through text messaging or phone call reminders from social and work peers. The use of alarms and pill boxes by patients prone to forgetfulness is also advised [17].

Findings showed that societal beliefs and norms shaped patients’ decision to refrain from adherence, because they are at variance with biomedical therapies for illnesses. The cultural path dependency theory provide insight into the phenomenon of norms shaping medication-taking [46]. The theory states that individuals although independent are governed by group norms and feedbacks from the wider society. Thus, societal socio-cultural norms that ‘incorrectly’ prohibit modern care is a feedback that can change individuals own actions to approximate the social environment [47]. We suggest the need for health providers to adopt cultural competence therapeutic approaches that take into account patients’ beliefs, values and norms in administering medications [48]. Knowing the patient’s ethnographic profile including prevailing norms can be useful in providing constructive prescription counselling.

Health providers’ actions were shown to influence non-adherence. They failed to properly explain the dosage regime to the understanding of patients. Also, a patient-centred approach of involving patients in prescribing decisions and answering pertinent questions of concern was lacking. This worked out to compromise adherence. Indeed, medication use is not only limited to telling the patient how and when to take drugs, but also, supporting the patient to take drugs by considering her preferences, beliefs and concerns in making prescribing decisions [22]. Another provider side factor that influenced non-adherence was poor written instructions about the dosage regime. Patients admitted to being confused as they tried to interpret the dosage regime due to poor hand writing. To get around this, they devised their own dosage calculation which was incorrect leading to complications and discontinuation of the medication [49]. We suggest the need for health providers, particularly prescribers to improve on their writing quality such that it convey simple information across to the patient.

**Fig 1:**
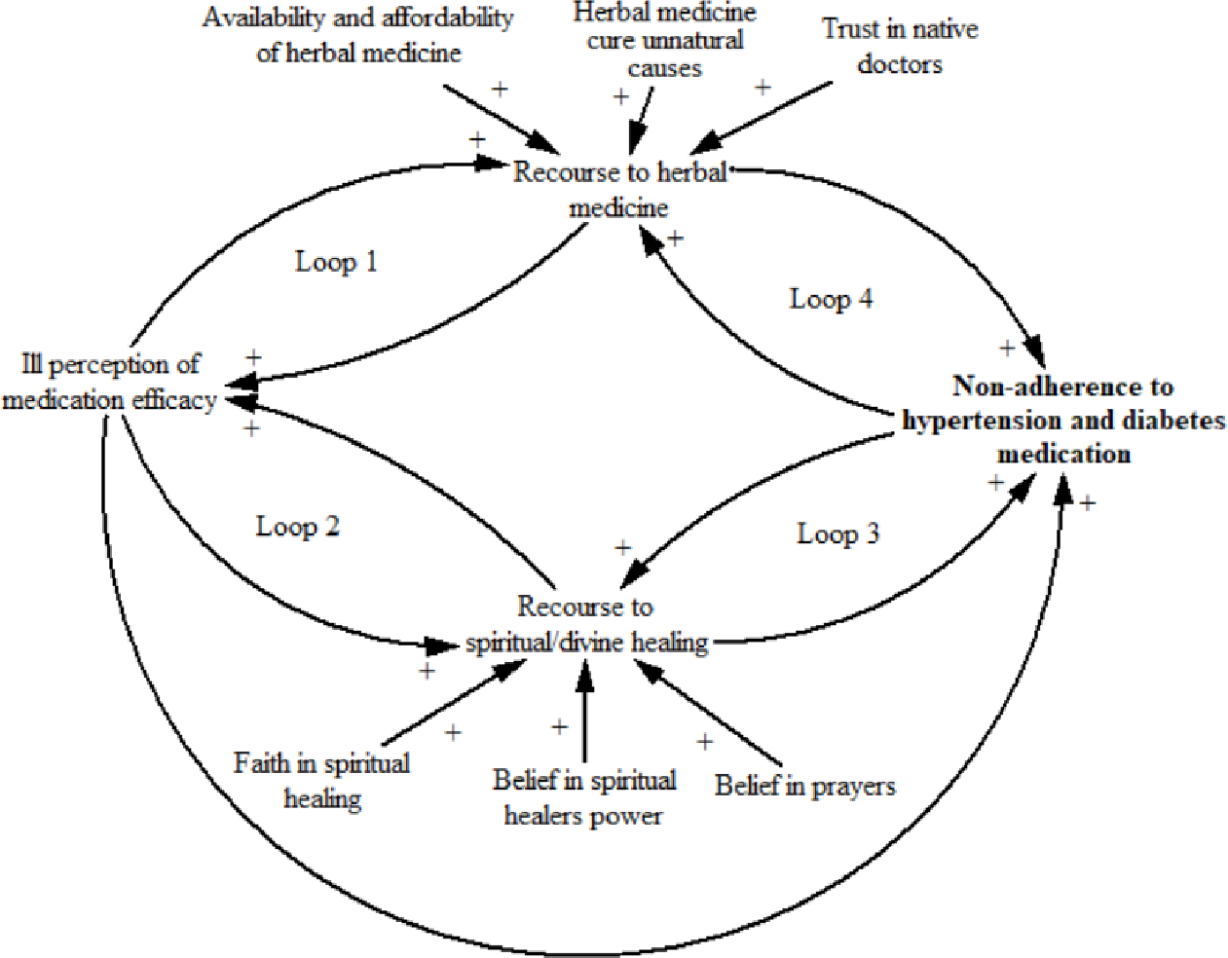
CLD of the interaction effect of perceived medication efficacy, recourses to herbal medicine and spiritual healing on non-adherence to diabetes and hypertension medication.

In summary, findings showed how and why non-adherence to diabetes and hypertension medication is variously influenced by all the eight factors. However, a closer look at the results shows that three of the factors, namely perceived medication inefficacy; recourse to herbal medicine and recourse to spiritual or divine healing strongly interacted to influence non-adherence. Thus, we drew on a causal loop diagram (CLD) (Fig 1), using Vensim software [50], to visualise and explain the interaction effect and feedback mechanisms of the three factors influence on non-adherence. As the Fig 1 shows, the more patients doubted the medication efficacy, the more they preferred herbal medicine, which in turn increases the perception that diabetes and hypertension medications do not have effective therapeutic effect. This reinforcing effect is illustrated by Loop 1. Similar reinforcing effect can be said of Loop 2 where ill perception of medication efficacy adds to the growing problem of recourse to spiritual or divine healing with a reversible consequence of increasing the burden of poor perception of the medications efficacy. In Loop 3, the more patients seek spiritual healing, the more likely is it that non-adherence increases which in turn produces a reinforcing effect on resource to spiritual healing. The same explanation can be given to Loop 4. Overall, recourses to herbal and spiritual healing ways increase with increasing ill-perception of the medications efficacy, all of which combine to strengthen non-adherence.

### Limitations

The study has limitations that should be noted. The fact that the study was conducted in one hospital is acknowledged as a limitation to generalisation of the findings; because non-adherence to medication is a complex issue that can vary by the practice setting. However, since the hospital studied is the largest referral tertiary care provider, findings overall may reflect likely outcome of replication of this study. The smallness of the sample size (49) and cross-sectional nature of the study are also acknowledged as limitations of the study.

### Conclusion

Medication non-adherence is a growing problem forestalling the management of chronic hypertension and diabetes [28]. Yet a comprehensive understanding of the causes of non-adherence has so far been given limited scholarly attention. Within the Ghanaian context, it is evident that such causes are diverse and complex, and emanate from the individual and the micro environment. A recent Ghana Health Service report, ranked hypertension and diabetes among the top 15 causes of outpatient visits [51], suggesting that large volumes of medications are needed to restore health among affected patients. However, drug supplies no matter the quantity will make limited impact in averting mortalities if patients poorly adhere to prescription. Our findings, thus, sends an important message to policy makers to act swiftly with appropriate interventions to encourage adherence among patients.
